# CEM immediately after contrast-enhanced CT: a one-step staging of breast cancer

**DOI:** 10.1186/s41747-024-00440-6

**Published:** 2024-04-01

**Authors:** Antonietta Ancona, Michele Telegrafo, Rita Roberta Fella, Donato Iamele, Sebastiano Cantore, Marco Moschetta

**Affiliations:** 1 Section of Breast Imaging, Breast Care Unit, Santa Maria Hospital GVM-BA, Via Antonio De Ferrariis 22, Bari, 70124 Italy; 2 Breast Care Unit, University Hospital Consortium Policlinico of Bari, Piazza Giulio Cesare 11, Bari, 70124 Italy; 3 DIM, Interdisciplinary Department of Medicine, Aldo Moro University of Bari Medical School, Piazza Giulio Cesare 11, Bari, 70124 Italy

**Keywords:** Breast neoplasms, Contrast media, Mammography (contrast-enhanced), Neoplasms staging, Tomography (x-ray computed)

## Abstract

**Background:**

Contrast-enhanced mammography (CEM) is a promising technique. We evaluated the diagnostic potential of CEM performed immediately after contrast-enhanced computed tomography (CE-CT).

**Methods:**

Fifty patients with breast cancer underwent first CE-CT and then CEM without additional contrast material injection. Two independent radiologists evaluated CEM images. The sensitivity of CEM for detecting index and additional malignant lesions was compared with that of mammography/ultrasonography by the McNemar test, using histopathology as a reference standard. Interobserver agreement for detection of malignant lesions, for classifying index tumors, and for evaluating index tumor size and extent was assessed using Cohen *κ*. Pearson correlation was used for correlating index tumor size/extent at CEM or mammography/ultrasonography with histopathology.

**Results:**

Of the 50 patients, 30 (60%) had unifocal disease while 20 (40%) had multicentric or multifocal disease; 5 of 20 patients with multicentric disease (25%) had bilateral involvement, for a total of 78 malignant lesions, including 72 (92%) invasive ductal and 6 (8%) invasive lobular carcinomas. Sensitivity was 63/78 (81%, 95% confidence interval 70.27–88.82) for unenhanced breast imaging and 78/78 (100%, 95.38–100) for CEM (*p* < 0.001). The interobserver agreement for overall detection of malignant lesions, for classifying index tumor, and for evaluating index tumor size/extent were 0.94, 0.95, and 0.86 *κ*, respectively. For index tumor size/extent, correlation coefficients as compared with histological specimens were 0.50 for mammography/ultrasonography and 0.75 for CEM (*p* ≤ 0.010).

**Conclusions:**

CEM acquired immediately after CE-CT without injection of additional contrast material showed a good performance for local staging of breast cancer.

**Relevance statement:**

When the CEM suite is near to the CE-CT acquisition room, CEM acquired immediately after, without injection of additional contrast material, could represent a way for local staging of breast cancer to be explored in larger prospective studies.

**Key points:**

• CEM represents a new accurate tool in the field of breast imaging.

• An intravenous injection of iodine-based contrast material is required for breast gland evaluation.

• CEM after CE-CT could provide a one-stop tool for breast cancer staging.

**Graphical Abstract:**

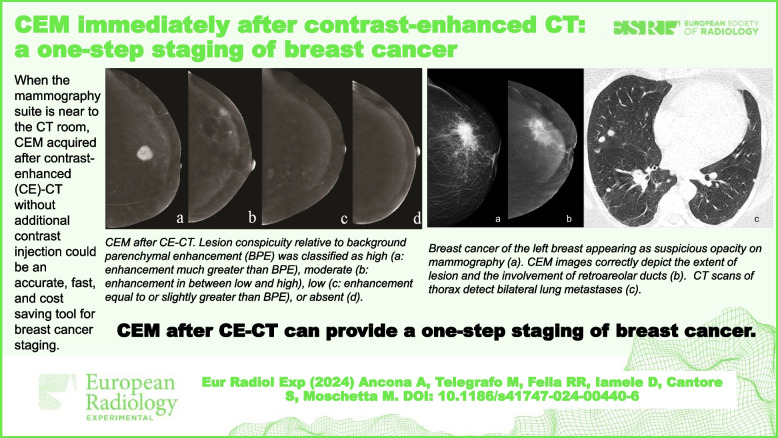

## Background

Contrast-enhanced mammography (CEM) is a promising breast imaging technique using iodinated contrast material and a pair of low- and high-energy images in order to highlight hyper vascular lesions [1–5].

A recent review and meta-analysis of CEM diagnostic performance demonstrated high performance in breast cancer (BC) detection, especially with joint interpretation of low-energy and recombined images and sensitivity and specificity values of 95% and 81%, respectively [6]. It represents an effective alternative to magnetic resonance imaging (MRI) in patients with contraindications to magnetic fields; in fact, both CEM and MRI are based on the same functional principles and evaluate the neoangiogenesis and contrast agent uptake of breast lesions. Furthermore, CEM has been reported to be a fast and less expensive imaging tool and to provide a higher positive predictive value and fewer false positive findings as compared to MRI [4–12].

In fact, except for high-risk patients who are particularly sensitive to radiation exposure and who require MRI surveillance, CEM is reported to be accurate for solving equivocal findings detected at conventional breast imaging, for the preoperative staging of breast cancer to evaluate the extent of disease, and for monitoring the response to neoadjuvant therapy [4, 6, 8–12]. In the field of preoperative staging, CEM shows lower cost and wider availability, reduces false-positive findings which can lead to additional biopsies, and patient anxiety as compared with MRI, even if it does not allow to evaluate the axilla or other local nodal stations [6–8].

In this scenario, patients undergoing contrast-enhanced computed tomography (CE-CT) for preoperative BC staging, the intravenous contrast material injection firstly used for CE-CT to detect distant metastases could be also exploited for subsequent CEM in order to evaluate the in-breast disease extent and, potentially, the contralateral breast. Only one study investigated the feasibility of performing CEM immediately after CE-CT without injecting additional contrast material and no difference in breast lesion detection was found if mammograms were acquired within 7 min or over 7 min after contrast material injection [13].

Similarly, the aim of our study was to evaluate the diagnostic potential of CEM performed immediately after contrast-enhanced CT for local staging of BC.

## Methods

### Patients

Our prospective study included 50 consecutive patients aged 55.9 ± 9.4 years (mean ± standard deviation), ranging 41–72 years, who were referred to the Breast Unit of our hospital in the timeframe between January 2020 and March 2022. All patients underwent mammography with digital breast tomosynthesis, breast ultrasonography (US), and core needle sampling confirming BC diagnosis. Written informed consent was obtained from all patients according to the Declaration of Helsinki.

We enrolled patients with indications to CE-CT basing on the 8th edition of the American Joint Committee on Cancer–AJCC manual for BC. Mammographic density was classified according to the 5th edition of ACR BI-RADS (American College of Radiology Breast Imaging Reporting and Data system) atlas [12]. Patients with renal failure, allergy to contrast agent, pregnancy, previous breast surgery, or breast implants were excluded.

Patients underwent first CE-CT and then CEM without additional contrast material injection. In the case of suspicious additional CEM findings, the patient underwent mammographic and US second look. If a suspicious correlated finding was found, US-guided or stereotactic core needle biopsy was performed.

### CE-CT

Examinations were performed using a 64-slice scanner (Somatom Sensation, Siemens Healthineers, Erlangen, Germany) before and after the intravenous injection of 1.5 mL/kg of iodine-based contrast material (iohexol, Omnipaque 350 mg I/mL, General Electric Healthcare, Milan, Italy) at a flow rate of 3 mL/s. CE-CT scans were performed with a triphasic technique in the arterial (40-s mean delay), venous (90-s mean delay), and delayed (140-s mean delay) phases, with patients in the supine position from the diaphragm dome to the pubic symphysis during the arterial and delayed phases and from the lung apices to the pubic symphysis during the venous phase. Technical parameters were: slice thickness 1.2 mm; reconstruction index 3 mm; pitch 1.25–1.75; tube rotation time, 0.5–0.75 s. Total body examinations were performed in all cases in order to obtain a systemic staging of BC according to TNM classification.

### CEM

CEM was performed after 6.8 ± 0.4 min (mean ± standard deviation) after contrast material injection using a Senograph Essential equipment (General Electric Healthcare, Milwaukee, WI, US). Low- and high-energy CEM images were acquired under automated parameters during breast compression without additional contrast material injection. In case of known unilateral BC, we acquired first craniocaudal and mediolateral oblique views of the affected breast and then the same views of the contralateral breast.

### Image analysis

Two independent radiologists with experience in breast imaging of 5 and 30 years and in CEM imaging of 5 and 10 years, respectively, evaluated CEM images and in particular the recombined contrast-enhanced images being aware that patients had histologically proven BCs and using the Breast Imaging Reporting and Data System CEM lexicon [14].

**Fig. 1:**
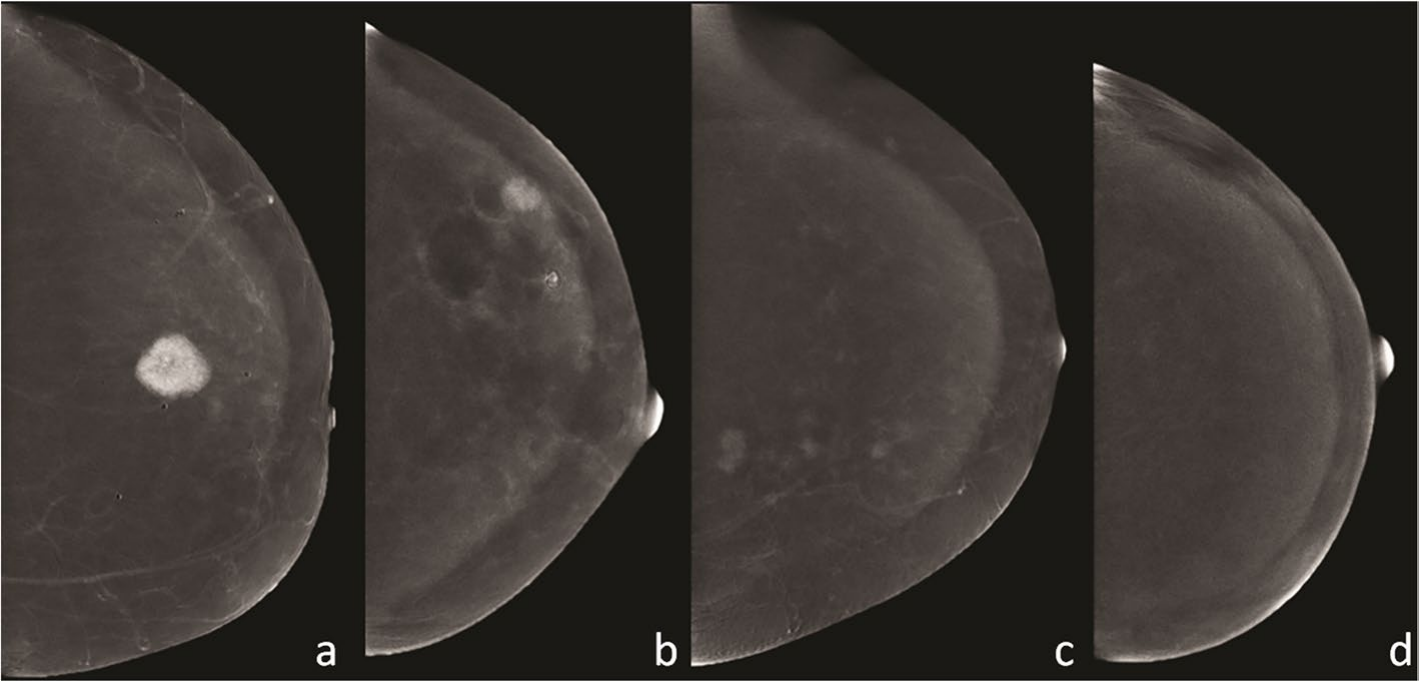
Classification of enhancement on contrast-enhanced mammography relative to background parenchymal enhancement (BPE): **a** high, enhancement much greater than BPE; **b** moderate, enhancement in-between low and high; **c** low, enhancement equal to or slightly greater than BPE; **d** no enhancement

Images were analyzed searching for enhancing areas with suspicious features. For each patient, a report indicating index tumor, its size and extent, and additional unilateral or contralateral lesions defining the disease as unifocal, multifocal, multicentric, or bilateral was provided. For each index tumor, the radiologists independently classified lesion conspicuity on CEM according to the above-mentioned lexicon [14]. Lesion enhancement/conspicuity relative to background parenchymal enhancement (BPE) was classified as high (enhancement much greater than BPE), moderate (enhancement in-between low and high PBE), or low (enhancement equal to or slightly greater than BPE) (Fig. 1).

### Histopathology

In all cases, the diagnosis of the index lesions and additional tumors detected by CEM and confirmed by second look ultrasound was performed by core needle biopsy. Immunohistochemical investigation was performed in all cases. Definitive postoperative histological examination was obtained in patients undergoing surgical therapy, and it served as the reference standard for correlating index tumor size and extent as detected by unenhanced breast imaging or CEM with the histological specimens. In all cases undergoing neoadjuvant therapy, the histological diagnosis was performed on specimens from core needle biopsies in the diagnostic setting, and they were excluded from the analysis of this correlation.

### Statistical analysis

We evaluated the sensitivity of CEM for detecting breast lesions which were known at unenhanced breast imaging (mammography/US) and the potential to detect additional malignant lesions, always having the histological findings as reference standard: histologically proven breast lesions at mammography or US plus histologically proven breast lesions at mammographic or US second look after CEM. The distribution of index tumors according to lesion conspicuity on CEM (high, moderate, low) was calculated. The McNemar test was used searching for any significant difference in terms of sensitivity by comparing unenhanced imaging and CEM. The interobserver agreement between the two radiologists in the overall detection of malignant breast lesions, in classifying index tumor according to the degree of CEM visualization/conspicuity, and in evaluating index tumor size and extent was calculated using Cohen *κ* coefficient, rating the agreement as follows: poor (*κ* ≤ 0.20), fair (*κ* = 0.21–0.40), moderate (*κ* = 0.41–0.60); (*κ* = 0.61–0.80), and near perfect (*κ* = 0.81–1.00). Pearson correlation coefficients were used for correlating index tumor size and extent as detected by unenhanced breast imaging or CEM with the histological specimens. Patients who underwent neoadjuvant therapy were excluded from this analysis based on the lack of a definitive histological evaluation of the extent of the disease before therapy.

## Results

### Lesions distribution and disease extent

Of the 50 patients, 30 (60%) had unifocal lesions, and 20 (40%) had multicentric or multifocal lesions; 5 out of 20 patients with multicentric lesions (25%) had bilateral involvement of the breast (Table 1). We found a total of 78 malignant lesions including 72/78 (92%) invasive ductal carcinomas and 6/78 (8%) invasive lobular carcinoma.

**Table 1:** Distribution of index lesions (detected by both unenhanced imaging and CEM) and CEM additional malignant lesions

Disease distribution	Patients (*n* = 50)	Lesions (*n* = 78)	Index lesions (*n* = 63)	CEM additional lesions (*n* = 15)
Unilateral, unifocal	30 (60%)	30 (38%)	30 (100%)	−
Unilateral, bifocal	11 (22%)	22 (28%)	13 (59%)	9 (41%)
Unilateral, multifocal	2 (4%)	6 (8%)	3 (50%)	3 (50%)
Unilateral, multicentric	2 (4%)	8 (10%)	5 (62%)	3 (38%)
Bilateral unifocal	3 (6%)	6 (8%)	6 (100%)	−
Bilateral multicentric	2 (4%)	6 (8%)	6 (100%)	−

*CEM* Contrast-enhanced mammography

Based on cancer histotype, biological characteristics, and TNM staging (12/50 patients, 50%, T1N0M0; 22/50, 44%, T2N1M0; 11/50, 22%, T4N2M0; and 5/50, 10%, T4N2M1), 12 out of 50 patients (24%) underwent neoadjuvant therapy while the remaining 38 patients (76%) underwent immediate surgical treatment (34/38, 89%, mastectomy; 4/38, 11% conserving surgery). Thirty-two out of 50 patients (64%) had axillary metastatic adenopathies, 11/50 (22%) had internal mammary metastatic adenopathies, and 5/50 (10%) had distant metastases involving lung (*n* = 3) or bone (*n* = 2).

### Comparison between unenhanced breast imaging and CEM

**Fig. 2:**
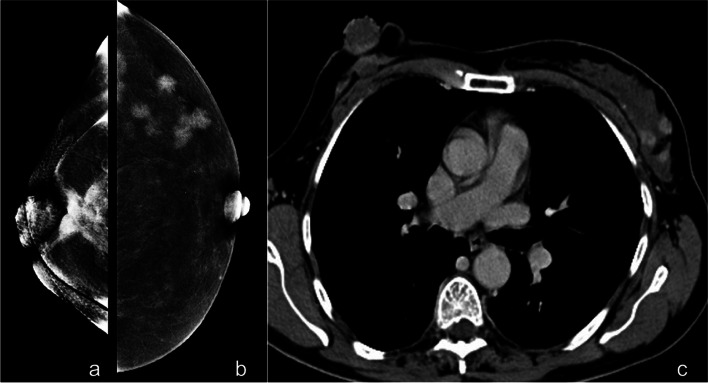
Patient with locally advanced cancer of the right breast (**a**) and multicentric contralateral lesions well recognized on CEM after CE-CT (**b**). Thoracic CE-CT scan of thorax showing bilateral breast disease (**c**). *CE-CT*, Contrast-enhanced computed tomography; *CEM*, Contrast-enhanced mammography

**Fig. 3:**
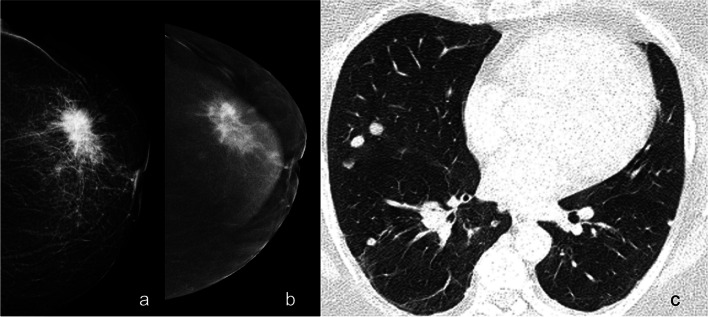
Breast cancer of the left breast appearing as a large spiculated mass on mammography (**a**). CEM correctly depicts the disease extent with involvement of retroareolar ducts (**b**). Thoracic CE-CT scans showing bilateral lung metastases (**c**). *CE-CT*, Contrast-enhanced computed tomography; *CEM*, Contrast-enhanced mammography

Comparing unenhanced breast imaging findings with overall histological findings (histologically proven breast lesions at mammography or US plus histologically proven breast lesions at mammographic or US second look after CEM), a sensitivity value of 81% was found (95% CI 70.27–88.82), while 100% was found for CEM (95% CI 95.38–100) (*p* < 0.001, McNemar test). Unenhanced imaging well recognized 63/78 lesions and missed 15 malignant lesions (false negatives) that were well recognized by CEM (Table 1; Figs. 2 and 3). However, in no case treatment options were changed based on these CEM additional findings neither from immediate surgery (*n* = 38) to neoadjuvant therapy or vice versa (*n* = 12) nor from conserving surgery (*n* = 34) to mastectomy (*n* = 4). The distribution of index tumors according to lesion conspicuity on CEM was as follows: high 58/78 (74%), moderate 20/78 (16%), low 0/78 (0%).

### Interobserver agreement and correlation between imaging and pathology

The interobserver agreement (*κ* value) between the two radiologists was 0.94 (95% confidence interval [CI] 0.81–1.00) for the overall detection of malignant breast lesions, 0.95 (95% CI 0.85–1.00) for classifying index tumor in terms of degree of CEM conspicuity, and 0.86 (95% CI 0.71–0.99) for evaluating index tumor size and extent.

Regarding index tumor size and extent, Pearson correlation coefficients were 0.50 (95% CI 0.27–0.59) for unenhanced breast imaging and 0.75 (95% CI 0.60–0.81) for CEM as compared with histological specimens (*p* = 0.002).

## Discussion

The choice of BC treatment is strongly influenced by the locoregional and systemic staging. Locoregional staging includes the evaluation of index tumor size and extent, the detection of additional foci of BC defining the disease as unifocal, multifocal, or multicentric, unilateral or bilateral, the involvement of skin or thoracic wall, and the involvement of axillary and internal mammary lymph nodes [5–11].

Contrast-enhanced breast MRI allows a reliable locoregional staging and represents the standard of reference for this purpose. Numerous studies have shown that MRI is superior to mammography and US for assessing the tumor size, yet there is still over- and underestimation in up to 15% of patients, and for detecting additional suspicious lesions, thus potentially leading to more extensive surgery. In particular, there is reported evidence that MR staging has a value in invasive lobular cancer, a histopathological BC subtype that is typically underestimated by mammography and US, and reduces reexcision rates in invasive lobular cancer, ranging from 11 to 18% [15, 16]. Moreover, the axilla is usually included in the field of view of breast MRI, and this allows a more global view of the axillae as compared with US, the detection of potentially abnormal lymph nodes, and the comparison of the bilateral axillae irrespective of patient body habitus. Moreover, it is more objective and less operator dependent than US [15–18].

With regard to the potential of CEM in assessing the BC extent, Jochelson et al. [1] demonstrated that CEM was significantly better than digital mammography in depicting the index tumor and that it was equal in sensitivity to MRI. In the ipsilateral breast, MRI depicted more additional malignant lesions than did CEM. In the contralateral breast, a single cancer was missed with both CEM and MRI. Additional findings appropriately changed treatment in 11 patients who underwent MRI compared with 8 patients who underwent CEM. However, MRI showed a higher false-positive rate compared with CEM. Fallenberg et al. [2] demonstrated equal sensitivity of CEM and MRI for both the index tumor and additional lesions. A third study by Kim et al. [17] including 84 women with newly diagnosed invasive BC and ductal carcinoma *in situ* found no significant difference in sensitivity to detect index cancers or additional lesions in either the ipsilateral or contralateral breasts between CEM and MRI, with similar changes in surgical management.

A study evaluating the role of CEM in presurgical planning [1] found that CEM depicted 98% of index lesions among 128 women with BC, missing a case of Paget disease and a parasternal lesion not included in the field of view; 67% of patients required additional biopsies based on CEM findings and had additional malignant lesions. CEM changed surgical management in 20% of patients, leading to mastectomy in 4%.

Therefore, CEM can be a viable alternative to MRI in preoperative staging, with accurate assessment of index tumor size, similar or possibly slightly inferior detection of additional sites of disease, and higher positive predictive value for detected additional lesions, although it does not enable the evaluation of the axilla or other local nodal groups [1, 5–7, 10].

With regard to size and extent of the index tumor, few studies have shown that tumor sizes measured with CEM ranged from 0.03 mm to 5 mm of the actual tumor size measured at the time of surgery [1]. Although Fallenberg et al. [2] showed that CEM was most highly correlated with surgical specimens, Cheung et al. [19] showed better accuracy with MRI than with CEM for tumor size. Recently, also Taylor et al. [20] demonstrated that CEM represents an accurate tool for BC staging but with a lower sensitivity as compared with MRI in a series of 59 patients.

In our study, we evaluated the feasibility of locoregional staging of BC by performing CEM immediately after CE-CT without the need for intravenous injection of additional contrast material. We selected a group of patients scheduled for CT staging who required a second level assessment by MRI or CEM. We tried to perform CEM within 7 min after contrast material injection to obtain a reliable enhancement of possible malignant breast lesions, even though Okada et al. [13] showed that also with longer time still allow for good breast CEM evaluation.

The evaluation of interobserver agreement between the two radiologists in the overall detection of malignant breast lesions, in classifying index tumor basing on the degree of CEM lesion conspicuity, and in evaluating index tumor size and extent allowed to evaluate whether the assessment of the CEM images was reliable without a loss of image quality and reproducible regardless of the different reader experience in the field of breast imaging. The interobserver agreement between the two radiologists in the overall detection of malignant breast lesions, in classifying index tumor basing on the degree of CEM visualization, and in evaluating index tumor size and extent was almost perfect. In particular, both the radiologists well recognized index tumors, and the degree of index tumor conspicuity on CEM was high or moderate without any loss of image quality. With regard to the index tumor size and extent, the measurement performed on CEM images proved high reliability with an interobserver agreement of 0.86 and showed a better correlation with histological specimens than unenhanced breast imaging. Moreover, a total of 15 additional malignant lesions were recognized by CEM as compared with mammography or US, although the treatment options did not change in all cases.

The best of our knowledge, only the above-mentioned study by Okada et al. [13] evaluated the feasibility of CEM immediately after contrast-enhanced CT without injecting additional contrast material. Authors considered two groups of patients based on the length of interval between contrast material injection that is less and more than 7 min. They found no significant difference between the two groups [13]. They found that the tumor opacification was not invalidated even when the interval between the contrast material injection for CT and the start of CEM was more than 7 min.

In our study, CE-CT also provided information about the systemic staging of BC. In particular, 32 out of 50 patients had axillary lymph node metastases, 11 out of 50 patients had internal mammary lymph node metastases, and 5 out of 50 patients had distant metastases involving lung in 3 cases and bone in 2 cases.

Fifteen additional CEM malignant findings occurred in our study. The absence of any impact on treatment planning can be explained with the particular setting of our feasibility study and probably with the relatively small number of the enrolled patients. In fact, among the fifteen additional CEM malignant findings, nine represented bifocal lesions and three multifocal tumors with the same scheduled surgical conservative treatment, while the remaining three lesions were detected in three patients already diagnosed with multicentric disease for whom a mastectomy had been already planned.

Comparing our results with those reported by Okada et al. [13], we note that CEM acquired immediately after CT without injection of additional contrast material could represent an accurate way for local staging of breast cancer with a 100% sensitivity for tumor detection and a significant correlation with histological findings in terms of tumor size and extent evaluation. Therefore, our results report a second experience in this field of breast imaging confirming the previous experience described by Okada et al. [13] but also adding a potential role of CE-CT for a one-step systemic staging of breast cancer.

Our study had some limitations mainly represented by the limited number of patients, the need of a specific condition in which the CEM suite is near to the CT room, the absence of intraindividual comparison between CEM and MRI findings in our sample, and the only qualitative assessment of lesion conspicuity on CEM images.

In conclusion, in patients who undergo CE-CT for systemic staging of BC, CEM acquired immediately after CT without injection of additional contrast material could represent an accurate, fast, and cost-saving tool for performing local staging of disease. Therefore, in a single time and with a single injection of contrast material, a local and contralateral staging of BC can be accurately performed by CEM while a regional (involvement of axillary and internal mammary lymph nodes) and systemic staging is obtained by CT. Further studies are needed in order to verify this preliminary data in larger series, in patients with diverse ethnicity or in case of different or longer CE-CT protocols used for BC staging.

## Data Availability

The relevant data have been included in the manuscript. The datasets used and/or analyzed during the current study are available from the corresponding author on reasonable request.

## Abbreviations

BC: Breast cancer
BPE: Background parenchymal enhancement
CE-CT: Contrast-enhanced computed tomography
CEM: Contrast-enhanced mammography
CI: Confidence interval
MRI: Magnetic resonance imaging
US: Ultrasound, ultrasonography
